# A Hybrid Ensemble Model Based on ELM and Improved AdaBoost.RT Algorithm for Predicting the Iron Ore Sintering Characters

**DOI:** 10.1155/2019/4164296

**Published:** 2019-01-17

**Authors:** Sen-Hui Wang, Hai-Feng Li, Yong-Jie Zhang, Zong-Shu Zou

**Affiliations:** School of Metallurgy, Northeastern University, Shenyang 110819, China

## Abstract

As energy efficiency becomes increasingly important to the steel industry, the iron ore sintering process is attracting more attention since it consumes the second large amount of energy in the iron and steel making processes. The present work aims to propose a prediction model for the iron ore sintering characters. A hybrid ensemble model combined the extreme learning machine (ELM) with an improved AdaBoost.RT algorithm is developed for regression problem. First, the factors that affect solid fuel consumption, gas fuel consumption, burn-through point (BTP), and tumbler index (TI) are ranked according to the attributes weightiness sequence by applying the RReliefF method. Second, the ELM network is selected as an ensemble predictor due to its fast learning speed and good generalization performance. Third, an improved AdaBoost.RT is established to overcome the limitation of conventional AdaBoost.RT by dynamically self-adjusting the threshold value. Then, an ensemble ELM is employed by using the improved AdaBoost.RT for better precision than individual predictor. Finally, this hybrid ensemble model is applied to predict the iron ore sintering characters by production data from No. 4 sintering machine in Baosteel. The results obtained show that the proposed model is effective and feasible for the practical sintering process. In addition, through analyzing the first superior factors, the energy efficiency and sinter quality could be obviously improved.

## 1. Introduction

**Figure 1:**
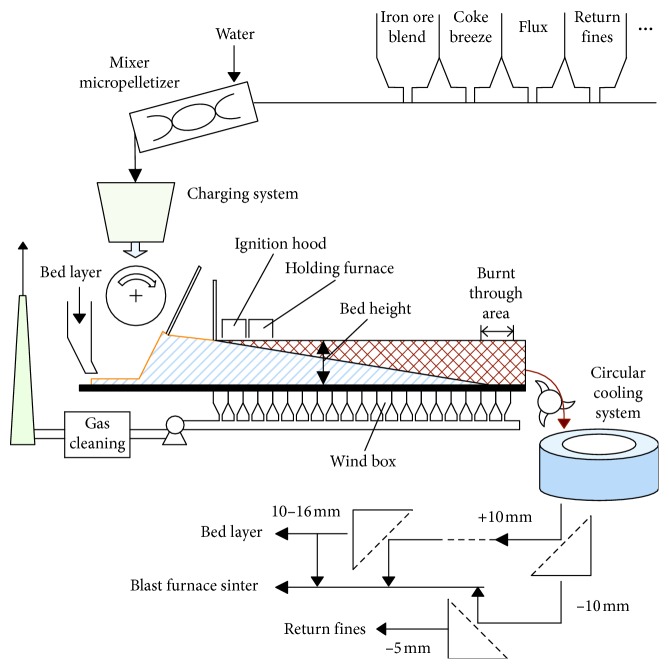
Schematic representation of iron ore sintering process.

The energy consumption of iron and steel enterprises in China is about 10% of the total energy consumption of the country. As a typical part of iron and steel making processes, the iron ore sintering process accounts for 10%∼15% of the energy consumption of an iron and steel enterprise.  Figure 1 shows the schematic view of the iron ore sintering process flow. A mixture of iron ore, fuel (coke breeze), flux (limestone/lime), return fines, and other additives is granulated with water in a rotary drum. These granulated mixtures are continuously charged together with bed layer material to form a thick bed of approximately 800 millimeters on a moving sinter strand. The sinter bed is ignited from the ignition hood, and the heat is drawn inside the bed under the action of air being sucked with the wind boxes under the strand. The holding furnace is used to maintain the heat absorbed by the surface of sinter bed sufficiently. The vertical sintering speed is controlled by strand speed and gas flow rate to ensure that the burn through occurs just prior to the end of the strand where sinter cake being discharged. The hot sinter cake is crushed followed by cooling in circular cooling system. The cold sinters are sieved and the undersize portion (the return fines, smaller than 5 mm) is delivered back to the raw materials feed system. The oversize portion is then transferred to the blast furnace and sieved the desired size (10–16 mm) used as the bed layer material.

In order to study the cause-effect relationships of the sintering process, expert systems [1–4], and prediction packages [5–8] have received particular attention due to the advantages of reliably representing nonlinear relations, which learn processes directly via historical data.

The solid fuel consumption, the gas fuel consumption, the burn-through point (BTP), and the tumbler index (TI) are the four characters that reflect the iron ore sintering performance. The location of BTP is the number of wind box which reached the highest temperature. The quality of sinter cake is determined by the BTP. For instance, if the BTP is located on the front of the normal point, the productivity of sinter bed is much lower than the designed level. On the other hand, if BTP is lagged behind, row material is less burned and thus, the the return fines increase. Scholars have made efforts to predict these characters. Wang et al. [9], for instance, have constructed the data-driven energy consumption model, taking the whole manufacturing process parameters as the variables. Chen et al. [10] have established the prediction model by BP neural networks, with accuracy of 91% for the comprehensive carbon efficiency. Wu et al. [11] have developed the BTP prediction model by using the support vector machines (SVM), taking the bed height, ignition temperature, and strand speed as the input variables. And Shang et al. [12] have developed the empirical dynamic model of BTP through the genetic programming. Kumar et al. [13] have developed the iron ore sinter properties prediction model through statistical analysis software system, with accuracies of the 79% for mean particle size (MPS), 91% for TI and 76% for reduction degradation index (RDI) prediction. Umadevi et al. [14] have developed the sinter quality analysis model to appraise how each key factors affect the sintering drum strength. In the actual sintering practice, the solid fuel and gas fuel consumption reflect the energy efficiency, the BTP reflects the process performance and TI reflects the property of the sinter products. Hence, the present work focuses the prediction models on these four characters.

In order to apply ensemble method to solve regression problems, Solomatine and Sherstha proposed AdaBoost.RT algorithm [15], where the letters R and T stands for regression and threshold, respectively. The absolute relative error (ARE) is used as the criterion to demarcate samples into correct and incorrect predictions. If the ARE of sample is less than the threshold *ϕ*, the predictor for this sample is remarked as the correct predictor; otherwise, it is regarded as incorrect. Such method is similar to project the regression problem into classification problem. The threshold *ϕ* do need to be selected initially, and it is a key factor that affected the performance of ensemble machines. According to Shreshtha and Solomatines' experiments [16], the ensemble model is stable while the value of *ϕ* is between 0 and 0.4. In order to determine the threshold value, Tian and Mao [17] presented a modified AdaBoost.RT algorithm by using a self-adaptive modification mechanism subjected to the change trend of the prediction error at each iteration. This approach has well performed in predicting the temperature of molten steel in ladle furnace, but the initial value of *ϕ* also needs to be manually fixed. Zhang et al. [18] established a robust AdaBoost.RT by considering the standard deviation of approximation errors to determine the threshold. The absolute error (AE) is used to demarcate samples as either well or poorly predicted in this approach. This method has well performed on the regression problems from UCI machine learning repository, but the relative factor *λ* should be optimized initially. In this study, a dynamically self-adjustable modifying the value of *ϕ* method is used instead of the invariable *ϕ* to improve the original AdaBoost.RT algorithm.

The present work proposes to optimize processing control by means of an ensemble predictor, which improves the energy efficiency of sintering process. Since there are many dependent or independent factors in the sintering production process, it is difficult and even impossible to develop mechanistic or detailed first-principle models that encompass all the factors, and it is clear that not the more input variables, the better performance of the model. The RReliefF algorithm is adopted to extract input attributes that are significant in terms of sintering characters. Then, the root mean square error (RMSE) is calculated in order to validate the approach of the attributes selection. The optimal parameter configuration of the proposed model is identified by the lowest value of RMSE. Finally, boosting-enhanced ELM with improved AdaBoost.RT is presented.

## 2. Soft Computing Algorithms and Attributes Selection

### 2.1. Extreme Learning Machine

An ELM [19] is an efficient learning algorithm for single hidden layer feedforward neural networks (SLFNs) used to solve the classification and regression problems. Compared with conventional neural networks, ELM is easy to use and theoretically achieve a globally optimum solution with much faster learning speed and good generalization performance. The weights between input layer and hidden layer are selected randomly and the output weights determined analytically during the learning process [20].

**Figure 2:**
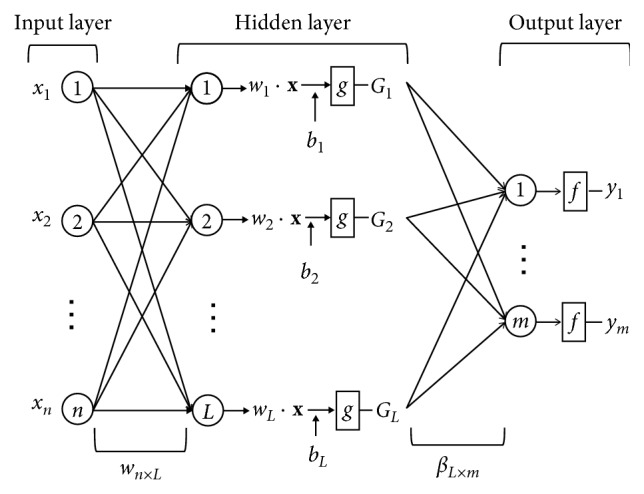
Illustration of the ELM network configuration.

A schematic of ELM network with *n* nodes in the input layer, *L* nodes in the hidden layer, and *m* nodes in the output layer is shown in Figure 2. The mechanism of ELM with multioutput is briefly depicted as follows.

For *N* arbitrary samples {(**x**_*i*_, **t**_*i*_)|**x**_*i*_ ∈ **R** ^*n*^, **t**_*i*_ ∈ **R** ^*m*^, *i*=1,2,…, *N*}, in which **x**_*i*_=[*x*_*i*_^1^, *x*_*i*_^2^,…,*x*_*i*_^*n*^]^T^ is the input vector and **t**_*i*_=[*t*_*i*_^1^, *t*_*i*_^2^,…,*t*_*i*_^*m*^]^T^ is the expected output. The output function of ELM with *L* hidden nodes and activation function **h**(*x*) is as follows:

(1) $$\begin{array}{c} f\left(x_{i}\right)=\overset{L}{\underset{j=1}{\sum }}\beta_{j}h_{j}\left(x_{i}\right)=h\left(x_{i}\right)\beta , \end{array}$$

where *β*_*j*_=[*β*_*j*1_, *β*_*j*2_,…,*β*_*jm*_]^T^ is the weight vector between hidden and output layers, **h**(*x*)=[*h*_1_(*x*), *h*_2_(*x*),…, *h*_*L*_(*x*)] is the output vector of hidden layer and denotes the feature mapping in ELM. The feature mapping **h**(*x*) is known to users with respect to the input *x*:

(2) $$\begin{array}{c} h\left(x\right)=\left[G\left(w_{1},b_{1},x\right),\ldots ,G\left(w_{L},b_{L},x\right)\right], \end{array}$$

where *w*=[*w*_1_, *w*_2_,…,*w*_*L*_]^T^ is the weight vector between input and hidden layers, *b*_*j*_ is the threshold of the *j*th hidden node, and *G*(*w*_*j*_, *b*_*j*_, *x*)=*h*(*w*_*j*_ · **x**_*i*_+*b*_*j*_) is the output of the *j* hidden node.

The objective of ELM is to calculate the weight vector *β* in minimizing both the output weights and the training errors:

(3) $$\begin{array}{c} \text{minimize}:\;L_{P_{\text{ELM}}}=\frac{1}{2}\left\|\beta^{2}\right\|+C\frac{1}{2}\overset{N}{\underset{i=1}{\sum }}\xi_{i}^{2},\\ \mathrm{subject}\;\;\mathrm{to}:h\left(x_{i}\right)\beta =t_{i}-\xi_{i},\;i=1,2,\ldots ,N, \end{array}$$

where *C* is a regularization coefficient, *ξ*_*i*_=[*ξ*_*i*_^1^, *ξ*_*i*_^2^,…,*ξ*_*i*_^*m*^]^T^ is the training error with respect to the input vector **x**_*i*_. The training of ELM is equivalent to solving the dual optimization problem according to the KKT theorem:

(4) $$\begin{array}{c} L_{D_{\text{ELM}}}=\frac{1}{2}\left\|\beta^{2}\right\|+\frac{C}{2}\overset{N}{\underset{i=1}{\sum }}\xi_{i}^{2}-\overset{N}{\underset{i=1}{\sum }}\alpha_{i}\left(h\left(x_{i}\right)\beta -t_{i}+\xi_{i}\right), \end{array}$$

where *α*_*i*_ is the Lagrange multiplier that corresponds to the *i*th training sample.

In order to simplify the computational complexity of ELM, two solutions can be obtained according to the scale of training samples by solving the dual optimization problem.

1. The training set is not huge: the **H** **H** ^T^ (size: *N* × *N*) is used in this case:

(5) $$\begin{array}{c} \beta =H^{\text{T}}{\left(\frac{I}{C}+HH^{\text{T}}\right)}^{-1}T, \end{array}$$

where

(6) $$\begin{array}{c} H={\left(\begin{array}{ccc} G\left(w_{1},b_{1},x_{1}\right) & \cdots & G\left(w_{L},b_{L},x_{1}\right)\\ \vdots & \cdots & \vdots \\ G\left(w_{1},b_{1},x_{N}\right) & \cdots & G\left(w_{L},b_{L},x_{N}\right) \end{array}\right)}_{N\times L}, \end{array}$$

and **T**=[**t**_1_, **t**_2_,…,**t**_*N*_]^T^ is the expected output vector of the training set. **H** in equation (6) is the hidden layer output matrix of the ELM network. Then, the final ELM output function is

(7) $$\begin{array}{c} f\left(x\right)=h\left(x\right)\beta =h\left(x\right)H^{\text{T}}{\left(\frac{I}{C}+HH^{\text{T}}\right)}^{-1}T. \end{array}$$

(2) The training set is huge: the **H** ^T^ **H** (size: *L* × *L*) is used in this case

(8) $$\begin{array}{c} \beta ={\left(\frac{I}{C}+H^{\text{T}}H\right)}^{-1}H^{\text{T}}T. \end{array}$$

In this case, the final ELM output function is

(9) $$\begin{array}{c} f\left(x\right)=h\left(x\right)\beta =h\left(x\right){\left(\frac{I}{C}+H^{\text{T}}H\right)}^{-1}H^{\text{T}}T. \end{array}$$

These two different methods to calculate the output weight vector can reduce the computational cost of ELM conveniently. For the small size of training data applications (*N* < *L*), the output of equation (7) can be used to rapidly increase the training speed, while the output of equation (9) always appear in the large scale data applications.

### 2.2. Improved AdaBoost.RT Algorithm

In order to determine the threshold *ϕ* value effectively, a novel improvement of AdaBoost.RT is proposed in the present work. We embed the statistics theory related to the regression capability of the weak learner into the AdaBoost.RT algorithm. A dynamically self-adjustable modifying the value of *ϕ* method is used instead of the invariable *ϕ*. For the training data sets with *m* samples (**x**_1_, *y*_1_),…, (**x**_*m*_, *y*_*m*_), in which *y*_*i*_ ∈ **R** is the output. The sample weights begin with uniform distribution initially:

(10) $$\begin{array}{c} D_{1}\left(i\right)=\frac{1}{m},\;i=1,2,\ldots ,m. \end{array}$$

Hence, the first training of the weak learning machine (WL) receives an equal weight for each sample.

In each subsequent iterations with index *t*+1(1 ≤ *t* < *T*), each sample weight *D*_*t*+1_(*i*) is determined by the fraction error *ε*_*t*_^*n*^ (*n* is power coefficient (e.g., linear, square, or cubic)) produced by the preceding iteration with respect to the sample (**x**_*i*_, *y*_*i*_):

(11) $$\begin{array}{c} D_{t+1}\left(i\right)=D_{t}\left(i\right)\times \left\{\begin{array}{c} \varepsilon_{t}^{n}\;\;{\text{ARE}}_{t}\left(i\right)\leq \phi ,\\ 1\;\;{\text{ARE}}_{t}\left(i\right)>\phi . \end{array}\right. \end{array}$$

The absolute relative error (ARE) for each training sample

(12) $$\begin{array}{c} {\text{ARE}}_{t}\left(i\right)=\left|\frac{f_{t}\left(x_{i}\right)-y_{i}}{y_{i}}\right|, \end{array}$$

is used as the criterion for demarcating samples into correct and incorrect predictions. In order to do so, a constant threshold value *ϕ* is introduced in the AdaBoost.RT algorithm. The predictions of the weak learner for those samples are considered as erroneous samples:

(13) $$\begin{array}{c} P_{t}=\left\{i\left|\left|\frac{f_{t}\left(x_{i}\right)-y_{i}}{y_{i}}\right|\right.>\phi \right\}. \end{array}$$

Hence, the error rate *ε*_*t*_ in equation (11) is given by

(14) $$\begin{array}{c} \varepsilon_{t}=\underset{i\in P_{t}}{\sum }D_{t}\left(i\right). \end{array}$$

The new weights *D*_*t*+1_(*i*) are normalized to ensure that *D*_*t*+1_ constitutes a probability distribution:

(15) $$\begin{array}{c} D_{t+1}\left(i\right)=\frac{D_{t+1}\left(i\right)}{\sum_{i}D_{t+1}\left(i\right)}. \end{array}$$

The final ensemble output hypotheses of AdaBoost.RT is

(16) $$\begin{array}{c} f_{\text{fin}}\left(x\right)=\frac{\sum_{t=1}^{T}\left(\log\left(1/\varepsilon_{t}^{n}\right)f_{t}\left(x\right)\right)}{\sum_{t=1}^{T}\left(\log\left(1/\varepsilon_{t}^{n}\right)\right)}. \end{array}$$

We propose a dynamically self-adjustable modifying the value of *ϕ* method to improve the AdaBoost.RT by embedding the statistics theory related to the regression capability of the weak learner into the training of ensemble predictor. The set of erroneous samples in our proposed method is given by

(17) $$\begin{array}{c} H_{t}=\left\{i\left|\left|\frac{f_{t}\left(x_{i}\right)-y_{i}}{y_{i}}\right|\right.>\frac{\sigma_{t}}{3\mu_{t}}\right\}. \end{array}$$

The *μ*_*t*_ and *σ*_*t*_ in equation (17) are the expected value and the standard deviation of the weak learners' predictions for the training set in the *t*th network. Then, the weak learners' error rate is

(18) $$\begin{array}{c} \varepsilon_{t}=\underset{i\in H_{t}}{\sum }D_{t}\left(i\right). \end{array}$$

Hence, the weights *D*_*t*+1_(*i*) are updated as

(19) $$\begin{array}{c} D_{t+1}\left(i\right)=D_{t}\left(i\right)\times \left\{\begin{array}{cc} \varepsilon_{t}^{n} & \left|\frac{f_{t}\left(x_{i}\right)-y_{i}}{y_{i}}\right|\leq \frac{\sigma_{t}}{3\mu_{t}},\\ 1 & \left|\frac{f_{t}\left(x_{i}\right)-y_{i}}{y_{i}}\right|>\frac{\sigma_{t}}{3\mu_{t}}. \end{array}\right. \end{array}$$

The proposed method overcomes the limitation suffered by the original AdaBoost.RT where the threshold value is set empirically. The critical threshold used in the boosting process becomes self-adaptive to the individual weak learners performance on the input data samples. Therefore, the proposed approach to improve the AdaBoost.RT algorithm is capable to output the final hypotheses in optimally weighted ensemble of the weak learners.

### 2.3. RReliefF-Based Feature Selection

The RReliefF algorithm [21] estimates the relevance of the attributes for solving a regression problem. RReliefF assigns a weight *W*[*A*] to each attribute *A* based on how well it distinguishes similar target values:

(20) $$\begin{array}{c} W\left[A\right]=\frac{P_{\left.\text{diff}\;\;C\right|\text{diff}\;\;A}P_{\text{diff}\;\;A}}{P_{\text{diff}\;\;C}}-\frac{\left(1-P_{\left.\text{diff}\;\;C\right|\text{diff}\;\;A}\right)P_{\text{diff}\;\;A}}{1-P_{\text{diff}\;\;C}}, \end{array}$$

where *P*_diff  *A*_ = *P* (different value of *A*| nearest instances), *P*_diff  *C*_ = *P* (different targets | nearest instances), and *P*_diff  *C*|diff  *A*_ = *P* (different targets | different value of *A* and nearest instances). The primary idea of RReliefF is that good attributes should separate instances with significantly different target values and not separate instances with close target values.

## 3. Hybrid Prediction Model of Iron Ore Sintering

In the present work, the RReliefF algorithm is used to select the input attributes for the ELM networks. The selected features influencing on the corresponding sinter characters can be ranked firstly through RReliefF. Then, we package the new superior features sequence into 5, 10, 15, and 20 and form as the input features set of the ELM network. Lastly, the root mean square error (RMSE) of the different input features set of the model are compared with each other, and the corresponding features set with the lowest value of RMSE is considered as the optimal variable group for the ELM networks. Therefore, the virtual model of sintering is optimized to predict the sintering characters.

**Figure 3:**
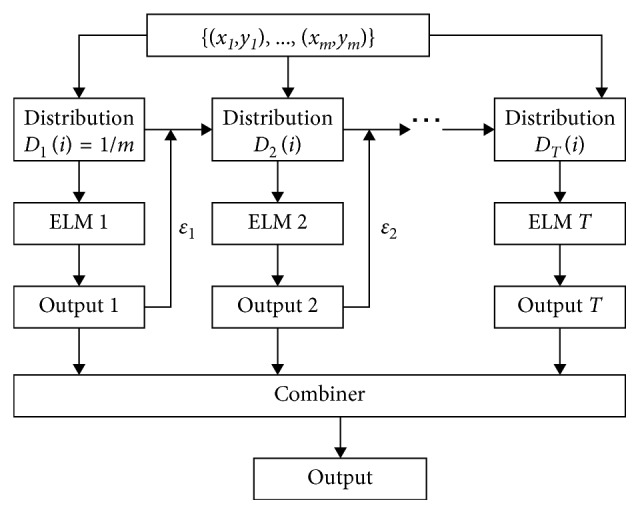
Ensemble ELM model using improved AdaBoost.RT algorithm.

Since the weight vector *w*_*j*_ between input nodes and hidden nodes and the threshold of *j*th hidden node *b*_*j*_ are selected randomly in the original ELM network, it does not have good stability and generalization capability for data with small sample size. With this in mind, we propose an ensemble model combined the improved AdaBoost.RT with ELM to improve the generalization capability of the original ELM algorithm. Here, the improved AdaBoost.RT is used as the ensemble method, and the ELM is the weak learner. The hybrid intelligent model is shown in Figure 3.

## 4. Experiments and Results

### 4.1. Variables and Data

A total of a year of operational data from no. 4 sintering machine in Baosteel are collected for modeling purposes. After removing the blank data, we introduce 3-sigma rule to deal with the abnormal value of measurement error. In applications, if the repeated measurement data satisfy

(21) $$\begin{array}{c} \left|x_{i}-\overset{¯}{x}\right|>3\sigma ,\;i=1,2,\ldots ,N, \end{array}$$

the *x*_*i*_ would be considered as an abnormal value and be rejected, where *x*_*i*_ is the *i*th measurement value, $\overset{¯}{x}$ is the average of all measurement values, and *σ* is the standard deviation of *x*_*i*_ sequence. This is the Pauta criterion of measurement error theory.

The total data available for modeling reduced to 270 data sets, which are used for training and testing with a network model. Descriptive statistics for the burdening indexes (*x*_1_ ~ *x*_5_), operating indexes (*x*_6_ ~ *x*_14_), chemical components (*x*_15_ ~ *x*_20_), and the four sinter characters (*Y*_1_ ~ *Y*_4_) are tabulated in Table 1. From these 270 sets of data, 220 are selected to serve as training set for ensemble ELM model. The residual 50 sets data recorded are used as testing set, which are never used during the training of the prediction model. In our experiments, all the input attributes are normalized into the range of [−1, 1], while the outputs are normalized into [0, 1]. The goodness of fit of the model is evaluated based on the values of MAE (mean absolute error), MRE (mean relative error), RMSE (root mean squared error), and Pearson correlation coefficient (*R*). The values of error parameters are calculated as follows:

(22) $$\begin{array}{c} \text{MAE}=\frac{1}{m}\overset{m}{\underset{i=1}{\sum }}\left|y_{i}-y_{i}^{\prime }\right|,\\ \text{MRE}=\frac{1}{m}\overset{m}{\underset{i=1}{\sum }}\frac{\left|y_{i}-y_{i}^{\prime }\right|}{y_{i}},\\ \text{RMSE}=\sqrt{\frac{1}{m}\overset{m}{\underset{i=1}{\sum }}{\left(y_{i}-y_{i}^{\prime }\right)}^{2}},\\ R=\frac{\sum_{i=1}^{m}\left(y_{i}-\bar{y}\right)\left(y_{i}^{\prime }-\bar{y^{\prime }}\right)}{\sqrt{\sum_{i=1}^{m}{\left(y_{i}-\bar{y}\right)}^{2}}\sqrt{\sum_{i=1}^{m}{\left(y_{i}^{\prime }-\bar{y^{\prime }}\right)}^{2}}}, \end{array}$$

where *y*_*i*_ is the actual value, *y*_*i*_′ is the predicted value, $\bar{y_{i}}$ is the average of actual values, $\bar{y^{\prime }}$ is the average of predicted values, and *m* is the size of testing set.

### 4.2. The Weightiness Sequence of the Attributes

**Figure 4:**
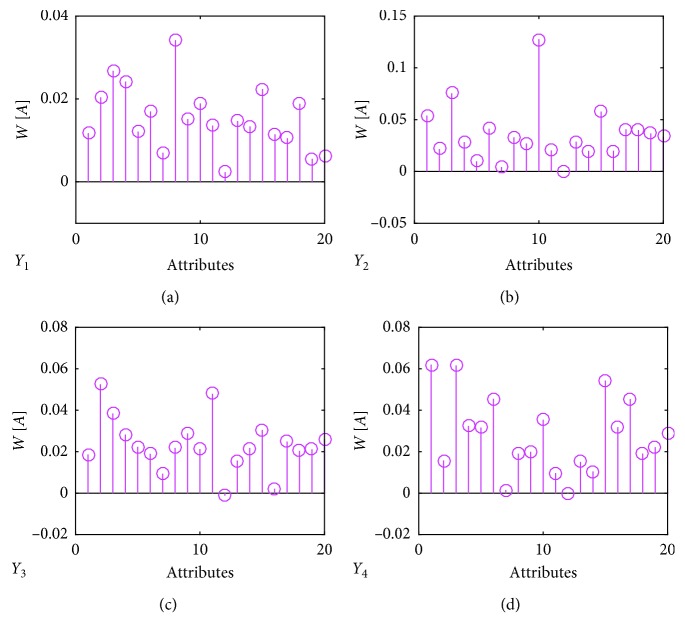
The weights for the 20 attributes as revealed by the RReliefF algorithm.

We applied the RReliefF algorithm to rank the attributes based on their merit scores. The weights are plotted in Figure 4. The 20 attributes for each character (*Y*_1_, *Y*_2_, *Y*_3_, and *Y*_4_) can be ranked as tabulated in Table 2. It can be found from Table 2 that the first superior factors of the four sintering characters are bed permeability, ignition density, dolomite, and limestone, respectively. The first five attributes in the weightiness order are considered as the first superior factor package to be the input into the ELM network prediction model and the RMSE is calculated. Then, the second five attributes in the weightiness sequence is combined with the first five attributes set and input into the ELMs network model until all the 20 attributes have been chosen. Lastly, RMSE of the above different attribute sets are compared with each other, and the corresponding attribute sets with the lowest value of RMSE is considered as the optimal attributes group for the ELM network prediction model.

### 4.3. Model Parameters Selection

**Figure 5:**
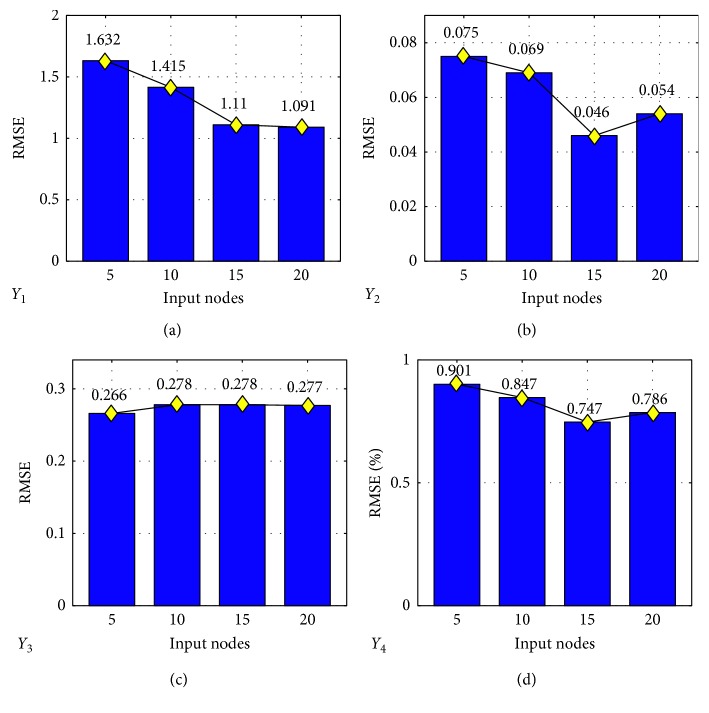
The average testing RMSE with different number of input nodes.

In the proposed prediction model framework, five user-specified parameters are need to be selected to achieve the best generalization performance. For ELM network, the sigmoid function *G*(**w**, *b*, **x**)=1/(1+exp(−(**w** · **x**+*b*))) is selected as the activation function. The cost parameter and hidden nodes are *C* and *L*, where *C* is chosen from the range {2^−24^, 2^−23^,…, 2^24^, 2^25^} and *L* is {10, 20,…, 1000}. The number of input attributes ranked by the RReliefF algorithm is considered as {5, 10, 15, 20}. Thirty trails of simulations have been conducted, and the performance of the (*C*, *L*) is verified using the average RMSE in testing. The best performed combinations of (*C*, *L*) are selected for each input nodes case as presented in Figure 5.

Figure 5 shows that RMSE of the testing sets are varied with the input nodes. To make the network efficiently calculate, the best performed of input nodes with the lowest RMSE has been chosen. Therefore, the input attributes of ELM network structure of solid fuel consumption is 20, the input attributes of gas fuel consumption is 15, the input attributes of BTP is 5, and the input attributes of TI is 15.

In addition, for the improved AdaBoost.RT-based ensemble ELM, the number of ELM networks needs to be determined. According to Occams Razor theory, simpler models may capture the underlying structure better and may have better predictive performance than excessively complex models which are affected by statistical noise. Therefore, the number of weak learners (*T*) need not be very large. In this paper, the number of ELM networks is set to be 5, 10, 15, 20, 25, and 30, and the optimal parameter is selected as the one which results in the best average RMSE in testing. Besides, the fraction error *ε*_*t*_^*n*^ should be optimized with *n*=1, 2, 3.  Table 3 shows the examples of setting both *T* and *ε*_*t*_^*n*^ for our simulation of the solid fuel consumption (*Y*_1_) with the best parameter combination selected according to Figure 5.

As illustrated in Table 3, the cubic fraction error performs better than the other two fraction error. The RMSE is less sensitive to the fraction error as long as the number of weak learners *T* is larger than 15. The ensemble model reaches the best performance with *ε*_*t*_^3^ and *T*=15, then we set the ensemble model with *T*=15 and *ε*_*t*_^3^ in the solid fuel consumption (*Y*_1_) experiment. The best performed combinations of user-specified parameters are selected for each sintering character as presented in Table 4.

### 4.4. Model Implementation

**Figure 6:**
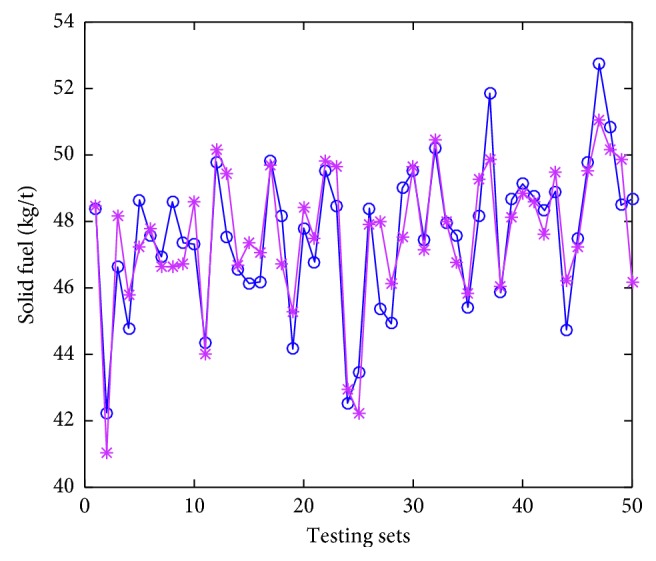
The comparison of measured values and predicted values of testing set for solid fuel consumption.

**Figure 7 fig7:**
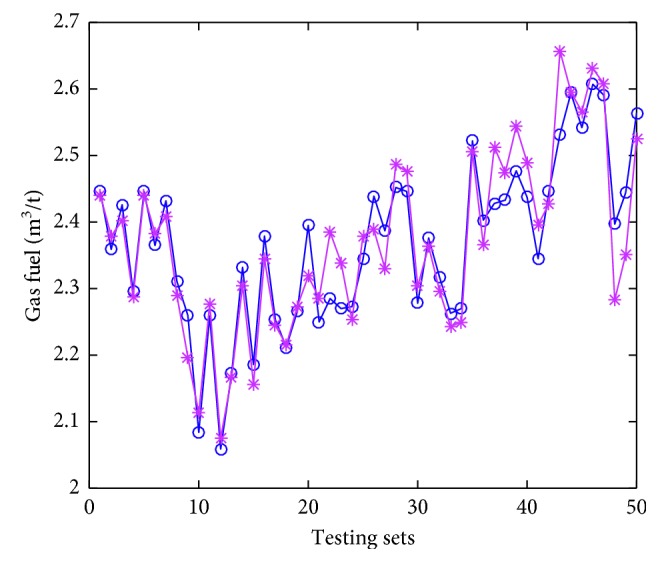
The comparison of measured values and predicted values of testing set for gas fuel consumption.

**Figure 8 fig8:**
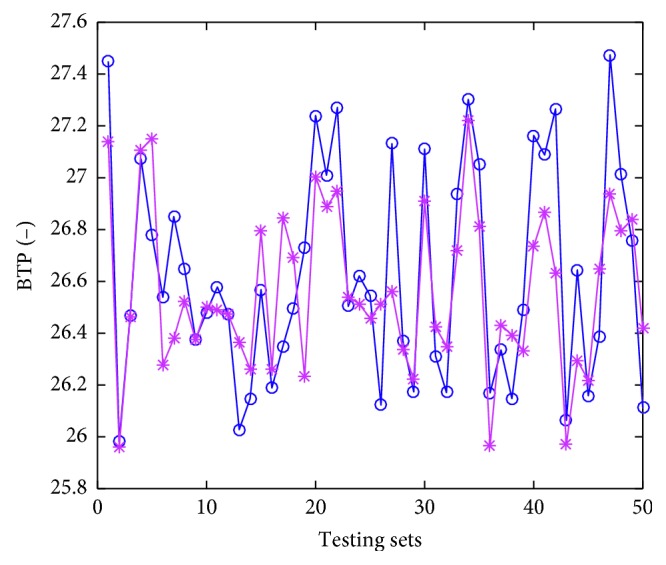
The comparison of measured values and predicted values of testing set for burning through point.

**Figure 9:**
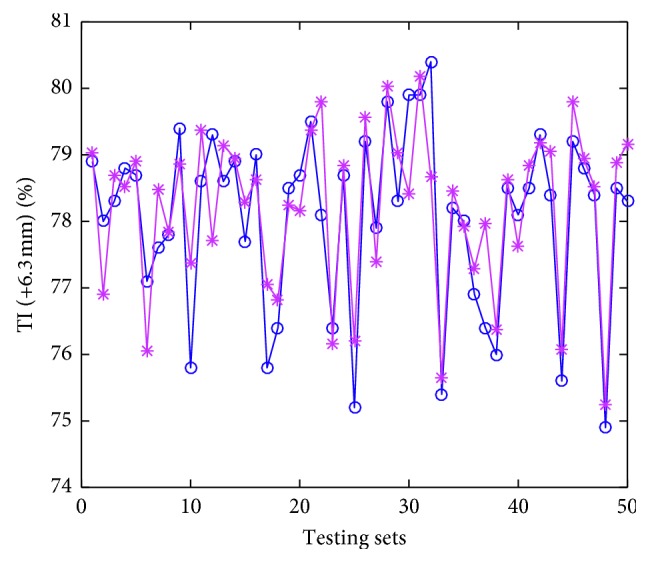
The comparison of measured values and predicted values of testing set for tumbler index.

Thirty trials of simulations have been conducted, and the averages serve as the “final results.” The testing errors are given in Table 5. The accuracy (predicting solid fuel consumption compared with real solid fuel consumption does not go beyond ±2 kg, that value for gas fuel consumption, BTP, and TI are ±0.1  m^3^, ±0.5, and ±1.5%, respectively) are calculated to describe the performance of the proposed model and also tabulated in Table 5. The predicted values denoted by asterisks, and actual values denoted by circles are displayed in Figures 6 [Fig fig7] [Fig fig8]–9.

As we can see in Figures 6 [Fig fig7] [Fig fig8]–9, the predicted values are pretty close to corresponding actual ones in most cases. From Table 5, the Pearson correlation between actual values and predicted values in gas fuel consumption experiment is 0.9383, and it is the highest in the four characters experiments. The results indicate that the accuracy of the hybrid ensemble prediction model based on ELM and improved Adaboost.RT is satisfied for the process production of iron ore sintering.

## 5. Application

**Figure 10:**
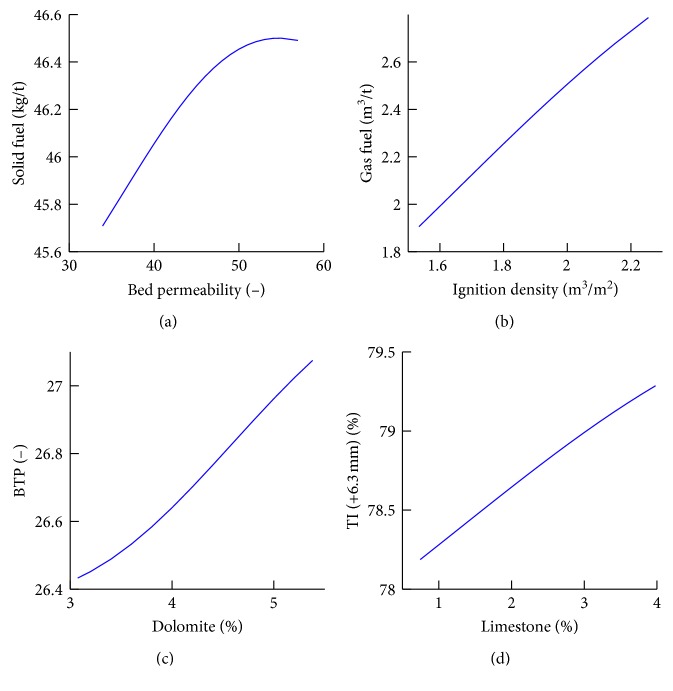
Sensitivity analysis of the first superior factor on sintering characters.

In order to figure out how the four sintering characters change with respect to the variation in input attributes, the sensitivity analysis of the first superior factor is conducted with the proposed model. The output value of the sintering character is calculated as the first superior factor is adjusted across its variation interval, while the other input attributes are kept fixed at their average values.  Figure 10 shows the influence of the four first superior factors on the four sintering characters.

The permeability of bed is a key factor that influences the sintering rate. The following equation is the bed permeability for the sintering machine [22]:

(23) $$\begin{array}{c} \frac{F}{WL}=\kappa {\left(\frac{P_{\text{v}}}{H}\right)}^{n}, \end{array}$$

where *F* denotes the air flow rate (Nm^3^/h), *W* denotes the pallet width (m), *L* denotes the strand length (m), *κ* denotes the bed permeability ((Nm^3^/h/m^2^)/(kPa/m)^*n*^), *P*_v_ denotes the suction pressure (kPa), *H* denotes the bed height (m), and *n* is an empirical factor (0.55∼0.65). Hence, high bed permeability allows faster air flow through the bed and thus faster sintering. An increase in bed permeability accelerates the heat front propagation speed through the bed. The fast air flow rate results in a lower thermal efficiency and higher energy consumption. Therefore, the solid fuel consumption increased with increase in bed permeability index.

The increase of ignition density results in the increase of gas fuel consumption. Ignition density define as the fuel gas (usually coke oven gas, COG) used during the igniting process. The purpose of ignition is to heat the sinter mixture that has been placed on the pallet to the semimolten state. The solid fuel in the mixture on the surface of the bed is ignited due to the igniting gas. Then, sintering is conducted from top to bottom under the action of suction of wind boxes. Thus, igniting is essential in iron ore sintering. The gas fuel consumption is increased as the ignition density increase.

The magnesium ferrite forms with the addition of dolomite in the sinter mixture and lowers the reducibility. Compared with CaO, MgO leads to an increase in the liquidus temperature of the melt phase. Therefore, the sintering period increases with dolomite. This results in BTP lagging behind; thus, BTP increased with increase in dolomite addition.

The strength of the sinter is dependent on the property and morphology of sinter. The high content of calcium ferrite in the iron ore sinter, in general, improves the tumbler strength of the sinter. The calcium ferrite phase increases with increase of the addition of limestone. Therefore, the TI increases with increase in limestone.

Through adjusting the first superior variables for the solid fuel, gas fuel, BTP, and TI, the optimization of the energy consumption and the control of the sinter quality can be realized efficiently. Thus, the shop floor operators can chose appropriate operating parameters by applying the rules derived from this hybrid prediction model objectively.

## 6. Conclusions

An integrated predictive model combined with feature selection and an ensemble method for sintering is proposed. RReliefF algorithm, as a mathematic method that ranks the sequence of the weightiness of lots of attributes in iron ore sintering system, can distinguish the superior influence parameters on energy consumption and sinter quality from the complicated factors. An improved AdaBoost.RT is proposed by using the statistics distribution of weak learners predicted values to dynamically determine the threshold. The virtual prediction model of the sintering process, which is combined the improved AdaBoost.RT with ELM network, has been achieved to simulate the sintering with the high coincidence using the production data in the steel making plant.

Adopting the ensemble ELM model, we can construct the solid fuel consumption prediction model with the prediction accuracy of 96%; the gas fuel consumption prediction model with the prediction accuracy of 96%; the BTP prediction model with the prediction accuracy of 94%; and the TI prediction model with the prediction accuracy of 90%. These are satisfied for the process production of iron ore sintering.

The improved AdaBoost.RT algorithm can promote the performance of the regression problems when the output value *y*_*i*_ ≠ 0 and the average value of predictions *μ* ≠ 0. If the true value of the sample comes to 0, the absolute relative error does not work. The proposed hybrid predicting frameworks can be programmed and validated conveniently in the Matlab platform.

## Figures and Tables

**Table 1:** Descriptive statistics of the variables.

Variables	Description	Unit	Minimum	Maximum	Average	Standard deviation
*x*_1_	Limestone	%	0.745	3.98	2.429	0.598
*x*_2_	Dolomite	%	3.074	5.381	4.208	0.434
*x*_3_	Quick lime	%	4.795	5.893	5.175	0.292
*x*_4_	Internal return fines	%	16.336	22.623	18.906	1.091
*x*_5_	Basicity	—	1.69	1.97	1.832	0.055
*x*_6_	Moisture rate	%	7.247	8.926	8.017	0.348
*x*_7_	Bed height	mm	653.444	816.063	768.691	35.457
*x*_8_	Bed permeability	—	33.935	56.957	45.276	3.575
*x*_9_	Strand speed	m/min	2.528	3.36	2.932	0.15
*x*_10_	Ignition density	m^3^/m^2^	1.534	2.255	1.824	0.141
*x*_11_	Ignition air-fuel ratio	—	4.98	6.321	5.124	0.182
*x*_12_	Insulation furnace temperature	°C	488.428	973.015	729.905	111.987
*x*_13_	Preheating air temperature	°C	229.296	328.856	294.24	14.022
*x*_14_	Exhaust fan negative pressure	kPa	12.616	19.483	16.886	1.141
*x*_15_	TFe	%	57.56	58.702	58.23	0.222
*x*_16_	FeO	%	7.376	10.498	8.806	0.573
*x*_17_	CaO	%	8.46	9.61	9.064	0.217
*x*_18_	MgO	%	1.258	1.78	1.483	0.1
*x*_19_	SiO_2_	%	4.545	5.282	4.952	0.122
*x*_20_	Al_2_O_3_	%	1.588	1.83	1.724	0.046
*Y*_1_	Solid fuel consumption	kg	40.963	53.971	47.223	2.304
*Y*_2_	Gas fuel consumption	m^3^	1.888	2.895	2.274	0.169
*Y*_3_	Burning through point	—	24.459	27.924	26.588	0.542
*Y*_4_	Tumbler index	%	74.2	84.1	78.424	1.5

**Table 2:** The weightiness sequence of attributes.

Attributes	*Y*_1_	*Y*_2_	*Y*_3_	*Y*_4_
*x*_1_	14	4	16	1
*x*_2_	5	14	1	15
*x*_3_	2	2	3	2
*x*_4_	3	12	6	7
*x*_5_	13	18	9	9
*x*_6_	8	5	15	4
*x*_7_	17	19	18	19
*x*_8_	1	10	10	14
*x*_9_	9	13	5	12
*x*_10_	6	1	12	6
*x*_11_	11	15	2	18
*x*_12_	20	20	20	20
*x*_13_	10	11	17	16
*x*_14_	12	17	11	17
*x*_15_	4	3	4	3
*x*_16_	15	16	19	8
*x*_17_	16	6	8	5
*x*_18_	7	7	14	13
*x*_19_	19	8	13	11
*x*_20_	18	9	7	10

**Table 3:** Performance of improved Adaboost.RT-based ELM with different fraction error *ε*_*t*_^*n*^ and *T* for solid fuel consumption (*Y*_1_).

*ε*_*t*_^*n*^	*T*					
	5	10	15	20	25	30
*n*=1	1.0901	1.0907	1.0871	1.0886	1.0907	1.0898
*n*=2	1.0932	1.0873	1.0868	1.0964	1.0873	1.0885
*n*=3	1.0878	1.0870	1.0853	1.0881	1.0899	1.0909

**Table 4:** Parameters of the proposed ensemble model.

Items	Sintering characters	Input attributes	(*C*, *L*)	(*ε*_*t*_^*n*^, *T*)
*Y*_1_	Solid fuel consumption	20	(2^2^, 810)	(*ε*_*t*_^3^, 15)
*Y*_2_	Gas fuel consumption	15	(2^2^, 800)	(*ε*_*t*_^3^, 25)
*Y*_3_	Burning through point	5	(2^2^, 800)	(*ε*_*t*_^2^, 20)
*Y*_4_	Tumbler index	15	(2^0^, 920)	(*ε*_*t*_^3^, 20)

**Table 5:** Summary of proposed model results.

Items	Index	MAE	MRE	RMSE	R	Accuracy (%)
*Y*_1_	Solid fuel	0.8649	0.0183	1.0835	0.8708	96
*Y*_2_	Gas fuel	0.0356	0.0149	0.0458	0.9383	96
*Y*_3_	BTP	0.2104	0.0078	0.2661	0.7827	94
*Y*_4_	TI	0.0057	0.0074	0.0074	0.8380	90

## Data Availability

The data used to support the findings of this study were supplied by Baosteel under license and so cannot be made freely available.

## References

[1] Fan X. H., Long H. M., Wang Y., Chen X. L., Jiang T. (2013). Application of expert system for controlling sinter chemical composition. *Ironmaking & Steelmaking*.

[2] Cores A., Muñiz M., Ferreira S., Robla J. I., Mochón J. (2013). Relationship between sinter properties and iron ore granulation index. *Ironmaking & Steelmaking*.

[3] Umadevi T., Karthik P., Mahapatra P. C., Prabhu M., Ranjan M. (2013). Optimisation of FeO in iron ore sinter at JSW steel limited. *Ironmaking & Steelmaking*.

[4] Vannocci M., Colla V., Pulito P., Zagaria M., Dimastromatteo V., Saccone M. (2016). Fuzzy control of a sintering plant using the charging gates. *Studies in Computational Intelligence*.

[5] Shigaki I., Narazaki H. (2010). A machine-learning approach for a sintering process using a neural network. *Production Planning & Control*.

[6] Zhang J.-h., Xie A.-g., Shen F.-m. (2007). Multi-objective optimization and analysis model of sintering process based on bp neural network. *Journal of Iron and Steel Research International*.

[7] Laitinen P. J., Saxén H. (2013). A neural network based model of sinter quality and sinter plant performance indices. *Ironmaking & Steelmaking*.

[8] Fan X.-h., Li Y., Chen X.-l. (2012). Prediction of iron ore sintering characters on the basis of regression analysis and artificial neural network. *Energy Procedia*.

[9] Wang J., Qiao F., Zhao F., Sutherland J. W. (2016). A data-driven model for energy consumption in the sintering process. *Journal of Manufacturing Science and Engineering*.

[10] Chen X., Chen X., She J., Wu M. (2016). Hybrid multistep modeling for calculation of carbon efficiency of iron ore sintering process based on yield prediction. *Neural Computing and Applications*.

[11] Wu X. F., Fei M. R., Wang H. S., Zheng S. B. Prediction of sinter burn-through point based on support vector machines.

[12] Shang X.-q., Lu J.-g., Sun Y.-x., Liu J., Ying Y.-q. (2010). Data-driven prediction of sintering burn-through point based on novel genetic programming. *Journal of Iron and Steel Research International*.

[13] Kumar V., Sairam S. D. S. S., Kumar S. (2016). Prediction of iron ore sinter properties using statistical technique. *Transactions of the Indian Institute of Metals*.

[14] Umadevi T., Naik D. K., Sah R., Brahmacharyulu A., Marutiram K., Mahapatra P. C. (2016). Studies on parameters affecting sinter strength and prediction through artificial neural network model. *Mineral Processing and Extractive Metallurgy*.

[15] Solomatine D. P., Shrestha D. L. Adaboost.RT: a boosting algorithm for regression problems.

[16] Shrestha D. L., Solomatine D. P. (2006). Experiments with Adaboost.RT, an improved boosting scheme for regression. *Neural Computation*.

[17] Tian H. X., Mao Z. Z. (2010). An ensemble ELM based on modified Adaboost.RT algorithm for predicting the temperature of molten steel in ladle furnace. *IEEE Transactions on Automation Science and Engineering*.

[18] Zhang L., Liu L., Bao S., Qiang M., Zou X. (2015). Transportation mode detection based on permutation entropy and extreme learning machine. *Mathematical Problems in Engineering*.

[19] Huang G. B., Zhou H. M., Ding X. J., Zhang R. (2012). Extreme learning machine for regression and multiclass classification. *IEEE Transactions on Systems, Man, and Cybernetics, Part B (Cybernetics)*.

[20] Liu X., Gao C., Li P. (2012). A comparative analysis of support vector machines and extreme learning machines. *Neural Networks*.

[21] Robnik-Šikonja M., Kononenko I. (2003). Theoretical and empirical analysis of ReliefF and RReliefF. *Machine Learning*.

[22] Nakano M. (2011). A differential analysis for the cost minimum operation of iron ore sintering machines. *ISIJ International*.

